# Text Book of Medical Jurisprudence, Etc.

**Published:** 1889-10

**Authors:** 


A TEXT-BOOK OF MEDICAL JURISPRUDENCE AND TOXICOLOGY. By John J. Reese, M. D., Professor of Medical Jurisprudence and Toxicology in the University of Pennsylvania, etc. Second edition, revised and enlarged. P. Blakiston, Son & Co., Philadelphia, 1889. 638 pp. Price, $3.

The subjects of legal medicine and toxicology have been conveniently condensed by Prof Reese, so a person can obtain the desired information on these subjects without wading through several volumes in a more extensive treaties.

This work has been adopted as a text-book in a great many of the best medical schools in the country. It is a standard, and being condensed, it is to be preferred, as a ready reference to more extensive works. Every physician should own a copy.

